# The effects of breastfeeding and formula feeding on the metabolic factors and the expression level of obesity and diabetes‐predisposing genes in healthy infants

**DOI:** 10.14814/phy2.15469

**Published:** 2022-10-05

**Authors:** Sahar Cheshmeh, Seyed Mostafa Nachvak, Niloofar Hojati, Negin Elahi, Neda Heidarzadeh‐Esfahani, Amir Saber

**Affiliations:** ^1^ Department of Nutritional Sciences, School of Nutritional Sciences and Food Technology Kermanshah University of Medical Sciences Kermanshah Iran; ^2^ Student Research Committee, School of Nutritional Sciences and Food Technology Kermanshah University of Medical Sciences Kermanshah Iran

**Keywords:** breastfeeding, diabetes, formula feeding, gene expression, infant, obesity

## Abstract

Diabetes mellitus (DM) and obesity are common illnesses characterized by glucose metabolism issues and excessive weight gain. Breastfeeding is the best way to feed a newborn up to 6 months old and it has been shown to reduce the risk of diabetes and obesity later in life due to its nutritional properties. The purpose of this study was to investigate the effects of breastfeeding, formula feeding, and formula‐plus breastfeeding (mix‐feeding) on the anthropometric indices, metabolic variables, and the expression level of obesity and diabetes‐predisposing genes of healthy infants. A total of 150 healthy infants were enrolled in this cross‐sectional study. All infants (aged 24 months) were divided into three groups based on the type of feeding, breastfeeding, formula feeding, and mix‐feeding. The anthropometric indices, glycemic indexes, lipid profile, and the expression levels of acetyl‐coenzyme A carboxylase beta (*ACACB*), brain‐derived neurotrophic factor (*BDNF*), liver X receptor α (*LXR‐α*), peroxisome proliferator‐activated receptor γ (*PPAR‐γ*), and phosphatase and tensin homolog (*PTEN*) genes were measured in all infants using reverse transcription‐polymerase chain reaction (RT‐PCR) method. The anthropometric indices including weight, height, head circumference, insulin, total cholesterol (TC), low‐density lipoprotein cholesterol (LDL‐C), and high‐density lipoprotein cholesterol (HDL‐C) were lower in the breastfeeding infants in comparison to other groups. As well, the expression level of the *ACACB* gene was significantly downregulated in breastfeeding infants, while the *PPAR‐γ* gene was significantly upregulated, but the expression levels of *LXR‐ α*, *PTEN* and *BDNF* did not change significantly across groups. Breastfeeding compared to formula feeding had positive effects on anthropometric indices, metabolic variables, and diabetes‐predisposing genes.

## Graphical abstract

This study aimed to investigate the effects of breastfeeding, formula feeding, and formula‐plus breastfeeding (mix‐feeding) on the anthropometric indices, metabolic variables, and the expression level of obesity and diabetes‐predisposing genes of healthy infants. A total of 150 healthy infants were enrolled in this cross‐sectional study. Breastfeeding compared to formula feeding had positive effects on anthropometric indices, metabolic variables, and diabetes‐predisposing genes.

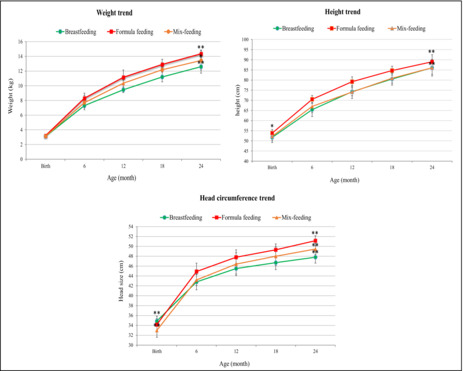

## 1 INTRODUCTION

Diabetes mellitus (DM) is a metabolic condition characterized by an imbalance in glucose metabolism. In recent years, the prevalence of DM had rapidly increased and is now estimated to be 8.8%, posing a worldwide health concern (Scheen & De Hert, 2007; Weisman et al., 2018). It is a multifactorial disorder with many acquisitive and non‐acquisitive variables such as genetics, age, obesity, diet, and lifestyle (Beckman et al., 2002; Weisman et al., 2018).

On the other hand, according to a World Health Organization (WHO) report, the prevalence of overweight and obesity in children has increased dramatically from 4% in 1975 to over 18% in 2016, and if this upward trend is not stopped, the global number of overweight or obese infants and children is expected to double in less than a decade (Freitas et al., 2019; WHO, 2016) Obese and overweight infants and children are more likely to become obese adults and suffer serious health issues such as type 2 diabetes, heart disease, asthma, stroke, diabetes, hypertension, dyslipidemia, and some forms of cancer at an earlier age (Friedemann et al., 2012; Lenz et al., 2009). Among all factors, it seems that lifestyle as a modifiable factor, plays a significant role in the etiology of diabetes. Different studies investigated the interactions between genes and lifestyle and showed that lifestyle and childhood eating habits may affect the DM predisposing genes and make a person prone to DM (Temelkova‐Kurktschiev & Stefanov, 2012).

Breastfeeding has several desirable effects on the child, mother, and health status of the community and is considered the best feeding way for infants throughout the first 6 months of life (James & Lessen, 2009; Pediatrics AAo, 2005). Numerous studies indicated the beneficial effects of breastfeeding on the prevention of chronic non‐communicable diseases such as autoimmune disease, cardiovascular diseases, cancer, chronic respiratory diseases, and diabetes (Kelishadi & Farajian, 2014). Besides, the link between breastfeeding and diabetes has been proved, and studies have shown that breastfeeding has protective effects against diabetes during childhood and adulthood (Kimpimäki et al., 2001; Norris & Scott, 1996; Virtanen et al., 2006).

Several studies have found that numerous beneficial compounds of breast milk cause epigenetic modifications in infants during their early life. These compounds have the ability to alter the DNA‐methylation process, which is crucial in the control of gene expression (Boddicker et al., 2016; Pathak & Feil, 2017). Also, these compounds are able to downregulate some genes such as *PTEN* (Li et al., 2015) and *ACACB* (Riancho et al., 2011) which can protect against diabetes. *PTEN* protein acts as a phosphatase, catalyzing the dephosphorylation of the inositol ring's 3′ phosphate in PIP3, resulting in the biphosphate product PIP2, which involves in several signaling pathways such as survival, cell proliferation, migration, and tumor suppression (Maehama & Dixon, 1998). Because insulin activity in the body is dependent on PI3K and PIP3 activation, it seems that mammalian *PTEN* protein may act in insulin or insulin‐like hormonal pathways and can regulate mammalian metabolism. Several studies have found that overexpression of the *PTEN* gene causes insulin resistance in insulin's downstream receptors. As a result, this gene might be a promising candidate for human autosomal dominant type II diabetes (Ogg & Ruvkun, 1998).

The *ACACB* gene encodes acetyl‐CoA carboxylase beta, which is involved in fatty acid biosynthesis and oxidation as the rate‐limiting step in mitochondrial fatty acid oxidation through carboxylation of acetyl‐CoA to malonyl‐CoA. Additionally, the ACACB gene has an important role in triglyceride synthesis, and previous studies have shown that the triglyceride content of muscle in obese people is higher than non‐obese people, and as an adaptation mechanism, the expression level of ACACB in adipose tissue decreased in response to increased body fat (Ma et al., 2011; Sinha et al., 2002; Wang et al., 2012). Several in vivo studies have shown that mice lacking the *ACACB* gene have a normal life, have higher insulin resistance, and fatty acid oxidation, and have lower fat mass (Abu‐Elheiga et al., 2001; Oh et al., 2005). As a result, because the *ACACB* gene plays an important role in energy metabolism in the body, it might be proposed as a marker gene associated with childhood obesity and other metabolic disorders such as diabetes (Li et al., 2017).

On the other hand, some genes such as *PPAR‐γ*, *LXRs*, and *BDNF* have been shown beneficial effects on glucose metabolism and diabetes (Krabbe et al., 2007; Murphy & Holder, 2000). *PPAR‐γ* is a ligand‐activated transcription factor that belongs to a superfamily of receptors and regulates gene transcription. (Dreyer et al., 1992) *PPAR‐γ* has shown a desirable effect on insulin resistance by activation of fatty acid transporters in adipose tissue, regulation of adipose tissue hormone release, and direct effects on glucose transport to tissues (Rangwala & Lazar, 2004). The *LXRs* gene has two isoforms that are important in carbohydrate and lipid metabolism (Edwards et al., 2002). *LXR‐α* is expressed exclusively in tissues involved in glucose and lipid metabolism, such as liver, kidney, adipose tissue, skeletal muscle, and intestinal tract, whereas *LXR‐β* is expressed in the majority of body tissues (Apfel et al., 1994; Song et al., 1994; Willy et al., 1995). *BDNF* is a neurotrophic factor that plays a role in the development, survival, and maintenance of neurons via the tropomyosin‐related kinase B high‐affinity receptor (*TrkB*) (Mattson et al., 2004). This neurotrophic factor affects several metabolic pathways by altering the hypothalamus or neurotransmitters that mediate food intake (Gray et al., 2006). Therefore, breast milk can reduce the risk of different metabolic‐related disorders and have long‐term effects on health status in adulthood through epigenetic changes and alterations in effective gene expression. The aim of this study was to investigate the effect of breastfeeding, formula feeding, and mix‐feeding on anthropometric indices, metabolic variables, and the level of expression of obesity and diabetes‐related genes in healthy infants.

## 2 MATERIALS AND METHODS

### 2.1 Study design and participants

**FIGURE 1:**
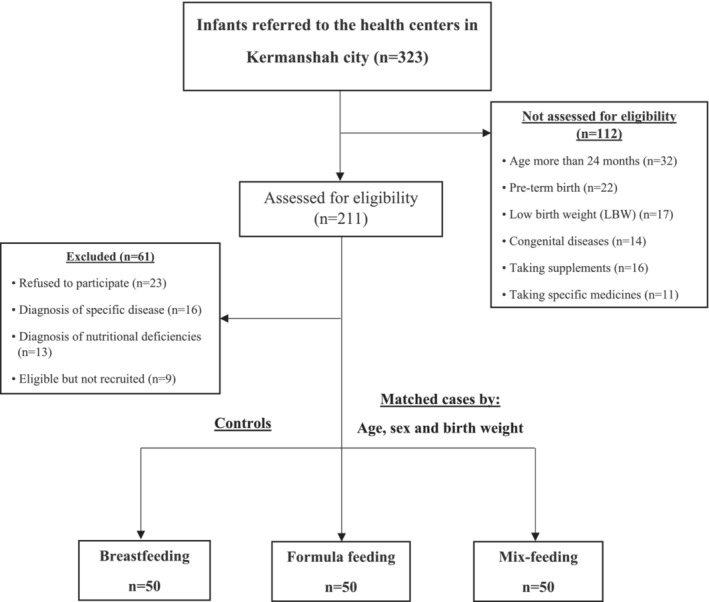
Flow chart illustrating the study population selection procedure, including infants' recruitment and exclusion criteria

This cross‐sectional study was conducted on healthy infants referred to Kermanshah city's health clinics in Iran. At first, all research procedures were clearly described for volunteered parents whose infants were eligible, and a formal written consent form was completed for all participants. After evaluating the inclusion and exclusion criteria, 150 healthy infants aged 24 months (boys and girls) were recruited among 323 infants referred to health clinics in Kermanshah city. All infants and their parents were healthy, and received breast milk or formula milk; if any metabolic disorders were detected in the infants or their parents before or during the study, they were excluded. All infants were divided into three groups: breastfeeding (*n* = 50) as the control group, formula feeding (*n* = 50), and mix‐feeding (*n* = 50) (Figure 1).

### 2.2 Measurements

Initially, the baseline characteristics of infants and their parents including age, sex, type, and duration of feeding were recorded by interviewing parents and using their valid ID cart. To establish the health status of infants anthropometric indices such as weight, height, and head circumference were measured using standard procedures and compared to WHO standard charts (Bammann et al., 2011). Furthermore, their prior anthropometric indices (from birth to 24th month age) data were recorded using their health record, and all information of infants was rechecked by their previously recorded data to reduce memory bias. In addition, parents' weight was measured with light clothing on a digital Seca scale with a precision of 0.1 kg, and their height was measured with a meter attached to the wall with a precision of 1 cm, and their body mass index (BMI) was calculated. For biochemical analysis, 5 ml of intravenous blood was collected from each infant after 8 h of fasting. The extracted serum samples were frozen at −80°C after centrifuging the blood samples for 15 min at 2000 *g*. Fasting blood sugar (FBS) was measured using an auto‐analyzer (Vita Laboratory) and the Pars Azmoon diagnostic kit (Parsazmun), and serum insulin was measured using the ELISA technique (Monobind). Moreover, triglyceride (TG), TC, and HDL‐C concentrations were measured enzymatically using the Pars Test kit (Parsazmun). The concentration of LDL‐C was also determined using Friedewald's formula, which is as follows: LDL‐C (mg/dl) = Total cholesterol (mg/dl) − HDL‐C (mg/dl) − Triglyceride (mg/dl)/5 (Krishnaveni & Gowda, 2015).

Blood samples from infants were collected in ethylenediaminetetraacetic acid (EDTA) coated vials for gene expression evaluation. Peripheral blood mononuclear cells (PBMC) were segregated through a density gradient centrifuge using the ficoll histopaque solution gradient (Ficoll‐paque, Miltenyi Biotec GmbH). The total RNA was isolated from PBMC using Trisol Reagent kit (YTzol pure RNA), and one μg of extracted RNA was utilized for complementary DNA (cDNA) synthesis using the Prime Script RT‐Reagent kit (Takara Bio Inc.) according to manufacturer's instructions. Specific primers were designed and purchased from Metabion Biotechnology Company (Metabion, steinkirchen) (Table 1). Data were normalized to 18 s rRNA gene expression level as the housekeeping gene by 2^−∆∆Ct^ method. All reactions were performed in triplicate and each experiment included a negative control.

**TABLE 1:** Primers sequences for RT‐PCR amplification

Gene name and symbol	Sequence (5՛→3՛)	Amplicon size (bp)	TM	
			*F*	*R*
*BDNF*	F: 5‐GGCTTGACATCATTGGCTGAC‐3՛	79	61.6	62.9
	R: 5‐TGTGCAGTGTGAGAAAGGCTT‐3՛			
*ACACB*	F: 5‐CAAGCCGATCACCAAGAGTAAA‐3՛	79	60.3	61.3
	R: 5‐CCCTGAGTTATCAGAGGCTGG‐3՛			
*PTEN*	F: 5‐CAAGATGATGTTTGAAACTATTCCAATG‐3՛	100	60.5	60.6
	R: 5‐CCTTTAGCTGGCAGACCACAA‐3՛			
*LXR‐α*	F: 5‐CCTTCAGAACCCACAGAGATCC‐3՛	83	61.7	62.4
	R: 5‐ACGCTGCATAGCTCGTTCC‐3՛			
*PPAR‐γ*	F: 5‐GATGCCAGCGACTTTGACTC‐3՛	186	61.1	62.4
	R: 5‐ACCCACGTCATCTTCAGGGA‐3՛			
*18s rRNA*	F: 5‐ACCCGTTGAACCCCATTCGTG A‐3՛	96	61.6	62.1
	R: 5‐GCCTCACTAAACCATCCAATCGG‐3՛			

Abbreviations: *ACACB*, acetyl‐coenzyme A carboxylase beta; *BDNF*, Brain‐derived neurotrophic factor; bp, base pair; F, forward; *LXR‐ α*, Liver X receptors α; *PPAR‐γ*, peroxisome proliferator‐activated receptor γ; *PTEN*, phosphatase and tensin homolog; R, reverse; TM, melting temperature.

### 2.3 Statistical analysis

All statistical analyses in this study were performed using the SPSS 23 software sciences (SPSS Inc., version 23.0). The Kolmogorov–Smirnov test was used to determine the normality of variables. For comparing quantitative variables between groups, the Chi‐square test was utilized. The Kruskal–Wallis test was used to evaluate non‐normal data while the One‐way ANOVA test was used to compare quantitative normal variables. All data were collected from at least three independent experiments and were reported as mean ± SD for quantitative variables and frequency (percentage) for qualitative variables.

## 3 RESULTS

The current study included 150 infants of both genders (50.4% boys and 49.6% girls). All infants were divided into three groups based on their feeding method: breastfeeding (*n* = 50), formula feeding (*n* = 50), and mix‐feeding (*n* = 50), and all of them completed the study. There were no significant differences in the baseline characteristics of infants and their parents between groups (Table 2).

**TABLE 2:** Baseline characteristics and anthropometric indices of infants based on types of feeding

Variable (mean ± SD)^a^	Breastfeeding	Formula feeding	Mix‐feeding^b^	*p*‐value^c^
	(*n* = 50)	(*n* = 50)	(*n* = 50)	
Sex				
Boy	26	25	28	0.74
Girl	24	25	22	
Age‐birth (week)	38.16 ± 1.74	38.8 ± 1.31	38.58 ± 1.53	0.09
Age of mothers (year)	26.40 ± 5.54	28.64 ± 6.40	27.24 ± 5.88	0.06
Age of fathers (year)	32.30 ± 4.54	34.64 ± 6.40	33.24 ± 5.88	0.07
BMI of mothers	24.60 ± 3.41	27.64 ± 3.82	27.74 ± 4.20	0.22
BMI of fathers	26.28 ± 3.41	26.50 ± 3.50	25.84 ± 2.90	0.32
Birth weight	3.10 ± 0.34	3.10 ± 0.38	3.0 ± 0.46	0.27
24th‐month weight (kg)	12.64 ± 1.24	14.27 ± 0.61	13.35 ± 1.01	<0.0001
Birth height(cm)	50.1 ± 2.9	52.3 ± 2.1	49.5 ± 2.8	0.31
24th‐month height	86.52 ± 2.71	88.78 ± 2.32	86.4 ± 2.92	<0.0001
Birth head circumference	42.8 ± 1.99	44.9 ± 1.4	43.2 ± 1.7	<0.0001
24th‐month head circumference	48.35 ± 1.48	50.20 ± 1.04	49.62 ± 0.99	<0.0001

^a^ Data are represented as means ± SDs and *n* (%) for continuous and categorical variables, respectively.

^b^ Mix feeding, breastfeeding plus formula feeding.

^c^ *p*‐values for comparison between groups using One‐way ANOVA test.

**FIGURE 2:**
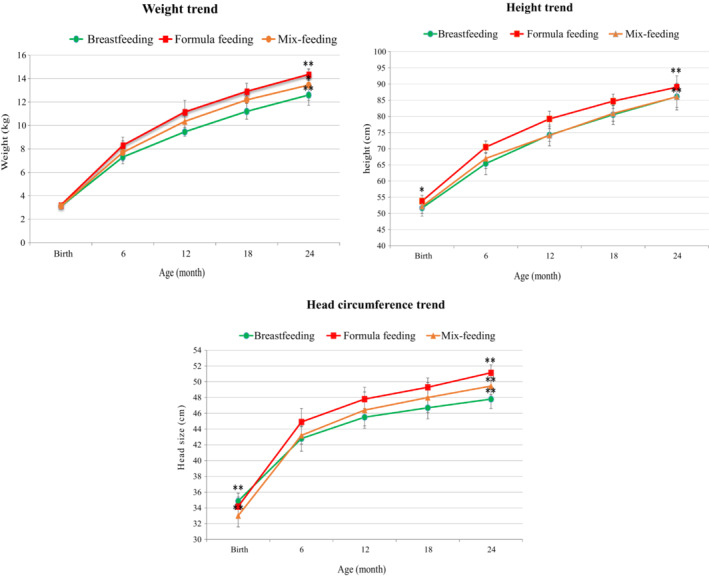
The comparison of the weight, height, and head circumference trends of infants from birth to the 24th month of life in three different groups (breastfeeding, formula feeding, and mix‐feeding). *p*‐value for comparison between groups was obtained using the one‐way ANOVA test. *p ≤ 0.05, **p < 0.01 as compared to the breastfeeding group

The comparison of the trend for weight, height, and head circumference of infants from birth to 24th month of age between the three groups revealed a significant difference between breastfeeding and formula feeding infants (Figure 2). As indicated in Table 2, there was a significant difference between the three groups for all anthropometric parameters, including 24th‐month weight (*p* < 0.0001), 24th‐month height (*p* < 0.0001), birth head circumference (*p* < 0.0001), and 24th‐month head circumference (*p* < 0.0001). Statistical analysis demonstrated a significant difference between breastfeeding and formula feeding groups for 24th‐month weight (*p* < 0.0001), birth height (*p* = 0.019), 24th‐month height (<0.0001), and 24th‐month head circumference (*p* < 0.0001). There was also a significant difference in 24th‐month weight (*p* = 0.015), birth head circumference (*p* < 0.0001), and 24th‐month head circumference (*p* < 0.0001) between the breastfeeding and mix‐feeding groups. Furthermore, except for birth weight and height, the differences in anthropometric variables between formula feeding and mix‐feeding infants were significant (Table 3; Figure 2).

**TABLE 3:** Comparison of anthropometric indices between groups based on type of feeding

Variable	Breastfeeding		Formula feeding
	Formula feeding^a^	Mix‐feeding^a^	Mix‐feeding^a^
Birth weight	*p* = 0.11	*p* = 0.30	*p* = 0.21
24th‐month weight	*p* < 0.0001	*p* = 0.01	*p* < 0.0001
Birth height	*p* = 0.01	*p* = 0.05	*p* = 0.08
24th‐month height	*p* < 0.0001	*p* = 0. 83	*p* < 0.0001
Birth head circumference	*p* = 0.60	*p* < 0.0001	*p* < 0.0001
24th‐month head circumference	*p* < 0.0001	*p* < 0.0001	*p* = 0.005

^a^ *p*‐values for comparison between groups using One‐Way ANOVA test.

Table 4 shows that, biochemical variables such as Insulin (*p* < 0.0001), TC (*p* < 0.0001), LDL‐C (*p* < 0.0001), and HDL‐C (*p* = 0.004) were significantly different across the three groups. In addition, the statistical analysis between breastfeeding and formula‐feeding infants showed that insulin (*p* < 0.0001), TC (*p* < 0.0001), LDL‐C (*p* < 0.0001), and HDL‐C (*p* = 0.001) were significantly different (Table 5). Besides, breastfeeding infants had a significantly lower median for TC (*p* < 0.0001) and lower mean for LDL‐C (*p* < 0.0001) compared to mix‐feeding infants. Only insulin (*p* < 0.0001) and TC (*p* < 0.0001) showed a significant difference between the formula feeding and mix‐feeding groups (Table 6).

**TABLE 4:** Biochemical variables of infants based on type of feeding

Type of feeding	Breastfeeding	Formula feeding	Mix‐feeding	*p*‐value^b^
Variable^a^	(*n* = 50)	(*n* = 50)	(*n* = 50)	
Blood glucose^c^	67.50 (58.75, 76.25)	60.50 (54.00, 70.00)	61.50 (54.75, 71.00)	0.05
Insulin	8.71 ± 3.42	13.13 ± 2.40	9.42 ± 3.46	<0.0001
HbA_1_c	5.16 ± 3.64	4.72 ± 2.19	4.89 ± 3.79	0.79
Triglyceride	75.94 ± 6.43	77.13 ± 5.91	74.72 ± 6.18	0.15
Total cholesterol^c^	120 (110.50, 129.25)	150.50 (136.25, 167.00)	130.00 (118.50, 142.50)	<0.0001
LDL‐C	39.78 ± 7.43	49.02 ± 5.22	48.7 ± 3.05	<0.0001
HDL‐C	47.78 ± 9.25	50.49 ± 9.34	48.84 ± 8.91	0.004

Abbreviations: HbA_1_c, hemoglobin A1c; HDL‐C, high density lipoprotein cholesterol; LDL‐C, low density lipoprotein cholesterol.

^a^ Data are represented as means ± SDs for continuous variables.

^b^ *p*‐values for comparison between groups using One‐way ANOVA test.

^c^ Data are represented as median (25th‐percentile, 75th‐percentile) and *p*‐values for comparison between groups using Kruskal–Wallis test.

**TABLE 5:** Comparison of biochemical variables between groups based on type of feeding

Type of feeding		Insulin^a^	Total cholesterol^b^	LDL‐C^a^	HDL‐C^a^
Breastfeeding	Formula feeding	*p* < 0.0001	*p* < 0.0001	*p* < 0.0001	*p* = 0.001
	Mix‐feeding	*p* = 0.30	*p* < 0.001	*p* < 0.0001	*p* = 0.09
Formula feeding	Mix‐feeding	*p* < 0.0001	*p* < 0.0001	*p* = 0.70	*p* = 0.09

Abbreviations: HDL‐C, high density lipoprotein cholesterol; LDL‐C, low density lipoprotein cholesterol.

^a^ *p*‐values for comparison between groups using One‐way ANOVA test.

^b^ *p*‐values for comparison between groups using Mann–Whitney *U*‐test.

**TABLE 6:** Gene expression of infants based on type of feeding

Variable^a^	Breastfeeding (*n* = 50)	Formula feeding (*n* = 50)	Mix‐feeding (*n* = 50)	*p*‐value^b^
*BDNF*	3.24 ± 2.84	2.77 ± 2.01	3.06 ± 2.27	0.61
*LXR‐α*	8.37 ± 4.64	8.29 ± 2.37	7.87 ± 1.80	0.70
*PPAR‐γ*	8.1 ± 2.03	6.54 ± 3.52	7.62 ± 1.42	0.007
*ACACB* ^c^	5.10 (2.65, 9.65)	19.26 (12.07, 40.50)	14.05 (9.37, 28.32)	<0.0001
*PTEN*	2.39 ± 2.42	2.17 ± 1.35	2.02 ± 1.97	0.63

Abbreviations: *ACACB*, acetyl‐coenzyme A carboxylase beta; *BDNF*, Brain‐derived neurotrophic factor; *LXR‐α*, Liver X receptors α; *PPAR‐γ*, peroxisome proliferator‐activated receptor γ; *PTEN*, phosphatase and tensin homolog.

^a^ Data are represented as median (25th‐percentile, 75th‐percentile) and *p*‐values for comparison between groups using Kruskal–Wallis test.

^b^ *p*‐values for comparison between groups using One‐way ANOVA test.

^c^ Data are represented as means ± SDs for continuous variables.

Based on Table 6, the median expression level of *ACACB* and the mean expression level of *PPAR‐γ* genes were significantly different between groups. Furthermore, there was a significant difference in the expression level of the *ACACB* gene between breastfeeding and the other two groups (formula feeding and mix‐feeding) (*p* < 0.0001). A similar comparison for *PPAR‐γ* gene expression was only significant between the breastfeeding and formula feeding groups (*p* = 0.008). The comparison of formula feeding and mix‐feeding groups showed a significant difference only in the expression level of the *PPAR‐γ* gene (*p* = 0.047) (Table 7).

**TABLE 7:** The comparison of gene expression between groups

Type of feeding	*ACACB* ^a^	*PPAR‐γ* ^b^
Breastfeeding		
Formula feeding	*p* < 0.0001	*p* = 0.008
Mix‐feeding	*p* < 0.0001	*p* = 0.19
Formula feeding		
Mix‐feeding	*p* = 0.07	*p* = 0.04

Abbreviations: *ACACB*, acetyl‐coenzyme A carboxylase beta; *PPAR‐γ*, peroxisome proliferator‐activated receptor γ.

^a^ *p*‐values for comparison between groups using Mann–Whitney *U*‐test.

^b^ *p*‐values for comparison between groups using Post‐Hoc test.

## 4 DISCUSSION

To the best of our knowledge, this is the first study that investigates how breastfeeding and formula feeding affect the expression level of *BDNF*, *LXR‐α*, *PPAR‐γ*, *ACACB*, and *PTEN* genes in infants. According to the findings of this study, anthropometric indices and biochemical variables such as 24th‐month weight, 24th‐month height, birth head circumference, 24th‐month head circumference, insulin, TC, LDL‐C, and HDL‐C were lower in breastfed infants than in others. Furthermore, *BDNF*, *LXR‐α*, and *PPAR‐γ* gene expression levels were higher in breastfed infants compared to others, but *ACACB* gene expression was lower.

Breastfeeding provides health benefits for both mothers and their children. Several studies have found that frequent breastfeeding reduces the incidence of type 2 diabetes, metabolic and cardiovascular disease, ovarian and breast cancer in women. On the other hand, infants who exclusively breastfeed, have a 22 to 24 percent lower risk of childhood and adolescent overweight than formula‐fed infants (Gunderson, 2008).

Numerous studies have shown that breastfeeding can prevent obesity and adiposity in adolescence and adulthood by improving anthropometric indices and biochemical parameters (Fenger‐Grøn et al., 2015; Horta et al., 2015; Marques et al., 2015). In this way, Gopinath et al. demonstrated that breastfeeding duration is significantly associated with lower BMI levels, and each month of increased breastfeeding is associated with an average reduction in BMI of about 0.04 and 0.03 kg/m^2^ among children aged 1–2 and 3–4 years, respectively. In addition, each month of increased breastfeeding duration is related with a 0.06 cm decrease in waist circumference in children aged 12–24 months (Gopinath et al., 2012). Moreover, different meta‐analyses and high‐quality studies have shown that breastfeeding can reduce the risk of diabetes and significantly decrease the incidence of overweight/obesity in both high‐income and low‐ or middle‐income countries in childhood by 13% (Arenz et al., 2004; Horta et al., 2015; Kramer & Kakuma, 2004). Similarly, our investigation showed that exclusive breastfeeding can help prevent childhood obesity by altering anthropometric indices.

Besides, Kimpimäki et al. (2001) revealed that short‐term exclusive breastfeeding and the early introduction of cow's milk‐based formula feeding might cause increasing symptoms of beta‐cell autoimmunity in infants who are genetically predisposed to type I diabetes. Cow's milk contains a variety of proteins and components, including α‐ and β‐lactoglobulin, Ig E, casein, and bovine serum albumin antibodies that stimulate the immune system in a damaging way against β‐cells and trigger type Ι diabetes in the first year of life (Hochwallner et al., 2014; Natale et al., 2004; Saukkonen et al., 1998; Xinias et al., 2019).

In addition, the results of a cohort study demonstrated that the infants who breastfed for less than 6 months had a higher risk of excessive weight gain at the age of 2 years than infants who breastfed for 6 months or more. These findings showed that the duration of breastfeeding is highly related to the risk of overweight so that breastfeeding for at least 6 months decreases the risk of weight gain in comparison to breastfeeding for no more than 1 month (Kalies et al., 2005).

The first few months of life are critical since development is rapid and the bodyweight doubles in 4–6 months (Ong et al., 2000; Plagemann et al., 2002). Therefore, in this period of life, the type of feeding has a key role in the healthy growth of infants and prevention of overweight and obesity in the future. Several studies have revealed that rapid weight gain during the first 4 months of infancy is associated with an increased risk of obesity by the age of seven (Papatesta & Iacovidou, 2013; Stettler et al., 2002).

The exceptional contents of human milk contribute to breastfeeding's unique role in preventing future overweight and obesity. Breast milk's nutritive and non‐nutritive components provide all of an infant's nutritional needs while also regulating their energy balance. Breast milk's unique bioactive substances, as well as its lower protein, fat, and insulin content, can regulate appetite, fat deposition, and metabolic responses (Gridneva et al., 2016; Kalies et al., 2005). Additionally, various components of breast milk, including as hormones, growth factors, neuropeptides, anti‐inflammatory and immune‐modulating substances, influence the growth, development, and function of the GI tract throughout early infancy (Gridneva et al., 2016). Human milk oligosaccharides (HMOs) are a unique component of breast milk that is not found in infant formula. These complex carbohydrates improve the gut microbiome and immune system of infants and recently have been suggested as a possible link between breastfeeding and lower obesity risk (Maessen et al., 2019).

Besides, human milk has a lower calorie density than formula milk, and breastfed children have better caloric self‐control than formula‐fed infants (Koletzko et al., 2010; Li et al., 2003; Ryan, 2007). According to several studies, consuming a high protein and nutrient‐dense diet lowers the basal metabolic rate and increases obesity by more than 10% in rats and humans (Daenzer et al., 2002; Koletzko et al., 2009). In this way, formula‐fed infants consume more protein than breastfed infants, which is associated with an increased risk of obesity via enhanced adipogenic activity and adipocyte differentiation (Koletzko et al., 2005). High protein consumption may reduce growth hormone (GH) release, resulting in reduced lipolysis of fat mass and effect on plasma free fatty acids (Koletzko et al., 2005; Marcus et al., 1994). Furthermore, feeding formula contains some growth‐promoting ingredients that accelerate children's growth in their early life and cause them to become overweight later in life (Ong & Loos, 2006; Stettler et al., 2002).

The most apparent difference between the two types of feeding is eating behavior and mother–child connection, since formula‐fed infants have longer intervals and different suckling habits than breastfed infants (Bosma et al., 1990; Koletzko et al., 2009; Mathew & Bhatia, 1989). On the other hand, breastfed infants can control the quantity, duration, and frequency of their meals based on their feeling of hunger and satiation (Koletzko et al., 2009; Priego et al., 2014).

Additionally, the type of feeding might affect an infant's insulin level and lipid profile. Our findings showed a significant difference in the insulin level and lipid profiles of infants depending on their feeding type and the exclusively breastfed infants had a lower insulin, TC, LDL‐C, and HDL‐C levels compared to other groups. The high protein content of the formula can stimulate the insulin release in formula‐fed infants and positively associated to the risk of overweight. Different investigations have found that formula‐fed infants have higher postprandial plasma insulin levels and a prolonged insulin response on day 6 of their life than breastfed infants (Lucas et al., 1981; Papatesta & Iacovidou, 2013). Higher protein consumption leads to an increased insulin and insulin‐like growth factor (IGF‐I) concentrations, which promotes adipose tissue deposition and the risk of overweight, obesity, and type 2 diabetes in the subsequent years (Putet et al., 2016).

In accordance with our findings, Hui et al. discovered that breastfeeding during the first 3 months can reduce TC, LDL‐C, and TG during adolescence. Similarly, our findings revealed that the difference in lipid profile between the mixed feeding and formula feeding groups was not statistically significant, with the exception of HDL‐C, which was lower in the mix‐fed group (Hui et al., 2019). On the contrary, Thorsdottir et al. demonstrated that the LDL‐C level in boy infants who breastfed for 48 months was significantly higher and the TC level was not significantly higher in 12 months' infants who breastfed at the ages of 2, 4, 6, 9, and 12 months. The HDL‐C level was significantly lower in infants who had been breastfed at the ages of 4 and 9 months. As well, in this study, the TG level was not significantly associated with breastfeeding (Thorsdottir et al., 2003) Likewise, Harit et al. demonstrated that exclusively breastfed infants had significantly higher TC and LDL‐C levels at 14 weeks and 6 months than mixed‐fed infants. Besides, TG level in breastfed infants were significantly higher at 14 weeks than in mixed fed infants. In the same way, Teller et al. revealed that breastfed infants had higher cholesterol and lipoprotein concentrations than formula‐fed infants (Teller et al., 2017). The higher lipid profile in exclusively breastfed infants appears to be advantageous for cognitive growth and lipid metabolism (Harit et al., 2008). Also, our results indicated that breastfeeding can significantly reduce TC and LDL‐C when compared to formula feeding and mix‐feeding. The discrepancy between our findings and those of other research might be attributed to differences in methodology, blood sample type (venous/capillary), time interval between last feeding and sampling, formula type, formula preparation (dilution), and genetic variations.

In addition, we found that the expression levels of *PPAR‐γ* and *ACACB* genes differed significantly between groups. In contrast, there was no statistically significant difference between the three groups for the *BDNF*, *LXR‐α*, and *PTEN* genes. Once the breastfeeding group was compared to the formula feeding and mix‐feeding groups, there were significant differences in *ACACB* gene expression (*p* < 0.0001). Also, the same comparison for *PPAR‐γ* gene expression was significant just for the breastfeeding and formula feeding groups (*p* = 0.008). The peroxisome proliferation‐activated receptor family is comprised of three isoforms: (1) *PPAR‐α*, *PPAR‐β/δ*, and *PPAR‐γ*, which differ in terms of physiological functions, ligand specificity, and tissue distribution. These isoforms play important roles in the regulation of genes involved in glucose and lipid homeostasis, cell differentiation, morphogenesis, and inflammatory response. The *PPAR‐γ* isoform is abundant in white and brown adipose tissue, the large intestine, and the spleen (Berger & Moller, 2002). Because of its importance in macronutrient metabolism, *PPAR‐γ* is a promising target for synthetic insulin sensitizers such as thiazolidinediones in the treatment of type 2 diabetes mellitus (Feige et al., 2006). According to some investigations, the activation of *PPAR‐γ* in adipocytes promotes the release of some beneficial adipocytokines on insulin sensitivity, such as leptin and adiponectin (Kintscher & Law, 2005).

Acetyl‐CoA carboxylase (ACAC) is an essential enzyme in fatty acid metabolism that is involved in the synthesis of malonyl‐CoA. *ACAC‐A* and *ACAC‐B* are two isoforms of ACAC that are encoded by genes on chromosomes 17 and 12, respectively. *ACAC‐B* is found mostly in the heart and skeletal muscle, where it catalyzes the carboxylation of acetyl‐CoA to malonyl‐CoA, which affects the quantity of fatty acid entering the mitochondria and fatty acid oxidation via modulating carnitine palmitoyltransferase‐1. As a result, *ACAC‐B* is critical in fatty acid synthesis and oxidation pathways, and any interruption in these pathways is associated with impaired insulin sensitivity and metabolic syndrome (MetS) (Abu‐Elheiga et al., 2000; Phillips et al., 2010; Riancho et al., 2011). Breastfeeding, as compared to formula feeding and mixed feeding, was found to decrease the expression level of the *ACAC‐B* gene in our study. Ma et al. showed that the *ACAC‐B* single‐nucleotide polymorphism (SNP rs2268388) is related to BMI in the general population and obesity in type 2 diabetes patients, and can affect some genes expression in adipose and hepatic tissue. They proposed that *ACAC‐B* plays an important role in obesity and may play a role in lipid metabolism abnormalities in type 2 diabetes (T2DM)‐associated nephropathy (Ma et al., 2013). Moreover, Abu‐Elheiga et al. demonstrated that *ACAC‐B* knock‐out mice are morphologically normal, develop at the predicted rate, and breed normally, despite having faster fatty acid oxidation rates and decreased fat and glycogen storage in adipose tissue and the liver, respectively (Abu‐Elheiga et al., 2001). The presence of an SNP in the *ACAC‐B* gene is associated to type 2 diabetic nephropathy susceptibility (T2DN) and it seems that targeting this pathway could be a potential therapy approach for diabetic nephropathy prevention (Tang et al., 2010). Besides, Riancho et al. revealed that different polymorphisms of the *ACAC‐B* gene affect energy metabolism in postmenopausal women, predisposing them to obesity and type 2 diabetes (Riancho et al., 2011). The *ACAC‐B* gene has been shown to have a significant role in the development of obesity and diabetes by lowering fat oxidation, and breastfeeding in infancy can help to minimize its negative consequences in adulthood. Also, the biological components in human milk have epigenetic effects through triggering some mechanisms including histone modification, DNA methylation, and chromatin remodeling. These active compounds have the ability to reduce or raise the expression of many critical genes in metabolic pathways, which may help to avoid weight gain and consequent non‐communicable illnesses later in life. As well, previous research has shown that breastfeeding can influence the key genes in the obesity pathway, decreasing the expression level of fat mass and obesity‐associated (*FTO*) and carnitine palmitoyltransferase IA (*CPT1A*) genes while increasing the expression level of the *PPAR‐α* gene in 5–6 month old infants (Cheshmeh et al., 2020). Likewise, in the current study, we investigated the effects of breastfeeding on other important genes in the obesity and diabetes pathways, such as *BDNF*, *LXR‐α*, *PPAR‐γ*, *ACAC‐B*, and *PTEN*, and noticed that breastfeeding significantly affected *PPAR‐γ* and *ACAC‐B* genes, confirming the epigenetic effects of human milk, which is deficient or absent in formula feeding.

This study had some limitations, including a small sample size and recall bias. By assessment of more infants, we could acquire more reliable and accurate outcomes. Since the duration and type of feeding were obtained by interview, there is a possibility for recall bias. Furthermore, due to the nature of cross‐sectional studies, we were unable to find cause‐effect links. It was not possible to establish a correlation between calorie intake, formula milk composition, and gene expression levels since the volume, calories, and components of formula powder were not available. In addition, due to financial constraints, we were unable to examine all obesity‐related genes in infants. The infants of both sexes, a satisfactory response and participation rate, and a standardized anthropometric assessment methodology were the study's strengths.

## 5 CONCLUSION

In conclusion, our findings show that anthropometric indices such as 24th‐month weight, 24th‐month height, birth head circumference, and 24th‐month head circumference are lower in breastfed infants than in formula and mix‐feeding infants. Additionally, some biochemical variables such as insulin, TC, LDL‐C, and HDL‐C were lower in breastfed infants than in the other two groups. In this study, human milk significantly decreased and increased the expression of the *ACAC‐B* and *PPAR‐γ* genes respectively. Also, there was no significant difference in the expression levels of the *BDNF*, *LXR‐α*, and *PTEN* genes between the three groups (breastfeeding, formula feeding, and mix‐feeding). Thus, breastfeeding can modify several obesity and diabetes‐predisposing genes and, when combined with other environmental variables, can be a potential effector in preventing weight gain in childhood. Overall, the recommendation of exclusive breastfeeding for the first 6 months of life is the best strategy for ensuring infants' healthy physical development and the prevention of communicable and non‐communicable diseases in adulthood. However, further research is needed to determine the impact of human milk on critical genes associated with obesity and diabetes, as well as the molecular pathways behind childhood and adult obesity.

## AUTHOR CONTRIBUTIONS

Nachvak SM, Saber A, designed the research, Cheshmeh S, Hojati N, Elahi N, Heidarzadeh‐Esfahani N, Nachvak SM conducted the research and contributed to manuscript revision, Cheshmeh S, Saber A analyzed the data and wrote the paper; Saber A had primary responsibility for the final content and all authors contributed to critical revisions and approved the final manuscript.

## FUNDING INFORMATION

This research did not receive any specific grant from funding agencies in the public, commercial, or not‐for‐profit sectors.

## CONFLICT OF INTEREST

The authors declare that there is no conflict of interest.

## ETHICS STATEMENT

This study was approved by the ethics committee of Kermanshah University of medical sciences with code No. IR.KUMS.REC.1397.069 and conducted in accordance with the Declaration of Helsinki.
